# Optimal Implementation of Prescription Drug Monitoring Programs in the Emergency Department

**DOI:** 10.5811/westjem.2017.12.35957

**Published:** 2018-02-22

**Authors:** Joshua W. Elder, Garrett DePalma, Jesse M. Pines

**Affiliations:** * University of California Davis School of Medicine, Department of Emergency Medicine, Sacramento, California; † The George Washington University School of Medicine and Health Sciences, Departments of Emergency Medicine and Health Policy & Management, Washington, District of Columbia

## Abstract

The opioid epidemic is the most significant modern-day, public health crisis. Physicians and lawmakers have developed methods and practices to curb opioid use. This article describes one method, prescription drug monitoring programs (PDMP), through the lens of how to optimize use for emergency departments (ED). EDs have rapidly become a central location to combat opioid abuse and drug diversion. PDMPs can provide emergency physicians with comprehensive prescribing information to improve clinical decisions around opioids. However, PDMPs vary tremendously in their accessibility and usability in the ED, which limits their effectiveness at the point of care. Problems are complicated by varying state-to-state requirements for data availability and accessibility. Several potential solutions to improving the utility of PDMPs in EDs include integrating PDMPs with electronic health records, implementing unsolicited reporting and prescription context, improving PDMP accessibility, data analytics, and expanding the scope of PDMPs. These improvements may help improve clinical decision-making for emergency physicians through better data, data presentation, and accessibility.

## INTRODUCTION

Due to the growing opioid epidemic in the U.S., there is widespread interest in using prescription drug monitoring systems (PDMP) to curb prescription drug abuse. PDMPs are statewide databases used by physicians, pharmacists, and law enforcement to obtain data about controlled-drug prescriptions, with the goal of detecting substance-use disorders, drug-seeking behaviors, and reducing patient risks of adverse drug events. While almost all U.S. states have PDMPs, they vary in design and implementation.^1^ In this paper, we review the history, evidence, and adoption of best practice guidelines in state PDMPs with a focus on how to best deploy PDMPs in emergency departments (ED). Specifically, we analyze the current PDMP model and provide recommendations for PDMP developers and EDs to help meet the informational needs of ED providers with the goal of better detection and prevention of prescription drug abuse.

## THE OPIOID CRISIS AND THE EMERGENCY DEPARTMENT

The U.S. accounts for roughly 80% of opioid use worldwide, and misuse – such as the recreational use of opioids – is a significant problem.^2^ Every 19 minutes in the U.S. someone dies from an unintentional drug overdose, the majority from opioids.^3^ From 1997 to 2007 the average milligram (mg)-per-year use of prescription per person of opioids in the U.S. increased 402%, from 74 mg to 369 mg. Meanwhile, an estimated seven million people above the age of 12 use opioids and other prescription medications for non-therapeutic purposes annually.^3^,^4^ These non-medical uses of opioids are linked to 700,000 ED visits yearly.^3^

Along with treating the consequences of opioid-related illness and overdose, EDs are often a location used by some patients as a source for opioid prescriptions.^4^ With limited time and no prior patient relationship, emergency physicians (EP) must make quick decisions balancing the provision of sufficient pain management against the potential for abuse and/or misuse. It is sometimes difficult to detect who might be seeking to misuse opioids. In one study, “classic” drug-seeking behaviors were relatively ineffective in identifying high-risk patients.^3^

### A Brief History of PDMPs and Their Effectiveness

PDMPs have been available for nearly 80 years. The first state PDMP was established in California (1939), followed by Hawaii (1943), Illinois (1961), Idaho (1967), New York (1970), Pennsylvania (1972), Rhode Island (1978), Texas (1981), and Michigan (1988).^5^ Early PDMPs were paper-based, recordkeeping systems used primarily to provide reports to law enforcement.^5^ By 1990 electronic key-punch databases enabled easier data dissemination via PDMPs, and pharmacists and clinicians began to use them.^5^ In 1996 the pharmaceutical OxyContin^TM^ was introduced; simultaneously, illicit prescription drugs doubled from 1994–1998.^5^ In response, Congress signed the Harold Rogers Prescription Monitoring Program grant into law in 2002, providing the first guidelines and funding for states to develop PDMPs.^5^ Since then, 49/50 states have now adopted PDMPs. (Missouri is the only exception).^6^

Since the inception of PDMPs, studies have assessed their impact on opioid prescribing and overdoses. Overall, the literature has been mixed. Some studies have found no relationship between PDMP implementation and outcomes; however, most studies evaluated paper- or faxed-based systems, with physicians receiving information days to weeks after the initial request.^4^,^7^ In one such study, 21% of the PDMPs evaluated were within their first years of operation or had only recently come online.^4^ This is significant because in states with new PDMPs, (Maine, New Mexico and Wyoming [which became operational in 2004–2005]), many physicians were not accessing or using data.^4^ This point is exemplified through Virginia’s PDMP, which was established in 2007 and was initially paper based.^8^ After moving to electronic and real-time reporting, data requests exponentially increased from 74,342 in 2009 to 433,450 in 2010.^8^

Another factor limiting the effectiveness of PDMPs is that each state has different policies and requirements for providers to use them. Few states mandate prescriber use and in those states that do not mandate use, compliance varies greatly.^7^ In this context, it is logical to assume that if prescribers do not access PDMP data, they cannot be effective.

## VARIATION IN PDMP DESIGN AND IMPLEMENTATION

A clear factor that leads to variation in observed effectiveness is that PDMPs are not all designed in the same way, particularly when it comes to their accessibility. Most are representative of separate and distinct technological and political environments at the time of their creation.^5^ According to the National Alliance for Model State Drug Laws study in late 2012, 38 state PDMPs are operated by a state health agency and six are operated under the aegis of law enforcement agencies.^9^ Additionally, 45 states monitor schedule II–IV controlled substances, 34 states grant authority to monitor schedule V substances, and 13 allow additional monitoring of drugs not listed on Drug Enforcement Administration schedules.^9^ Moreover, while several states require physicians to access patient PDMP data before prescribing controlled substances, the majority of states allow for voluntary participation among physicians.^9^ Finally, 25 states provide unsolicited, automatic reports of suspicious activity directly to law enforcement but only three (Delaware, Louisiana, and West Virginia) automatically send reports to multiple facilities including law enforcement, licensing boards, pharmacies, and prescribers.^9^

However, states have looked to update and reformat their PDMPs to better address the opioid crisis. For example, with funding from the Core State Violence and Injury Prevention Program, Oregon reformatted its PDMP to provide more appropriate data to its EPs.^10^ Under this new funding, PDMPs were designed to track schedule II–VI drugs prescribed within Oregon as well as providing physicians with access to the PDMP data of bordering states.^10^ Furthermore, pharmacies within Oregon were required to report prescription data within 72 hours of opioid dispensing.^10^ Such interstate sharing and tracking of all scheduled drugs has shown to provide safer patient care. Since implementation, Oregon has reported a 38% decrease in the rate of prescription opioid overdose as well as a 58% reduction in deaths related to methadone use.^11^ As sharing hubs such as those in Oregon, Michigan, Indiana and Ohio are established, EPs may be better equipped to successfully identify drug-seeking behavior.

PDMP design also leads to great variation in usability. For example, some information displayed is not always relevant or organized in a way that allows for EPs to answer specific clinical questions that fit into ED workflow. In some systems, frequent extraneous information is obtained simultaneously.^2^ Excessive data forces providers to search for relevant information, squandering valuable time.

Furthermore, clinician training on how to use and interpret PDMPs is often limited. Users are often left wading through mountains of patient data seeking to piece together a complete picture. One study surveyed physicians and nurses from diverse specialties after PDMP use and found that practitioners lacked guidance on data interpretation.^12^ In EDs time is a valuable resource and, unfortunately, the complexity of some PDMPs limits their usability. For example, in the current structure, PDMPs have experienced growing compliance issues secondary to their difficulty of use. In certain states, physicians are required to register with their PDMP via a notarized medical license and government identification.^13^ Password protocols exacerbate issues with PDMP accessibility. Often physicians are required to meet excessive requirements for password security only to find that within a short time their password has expired and the process starts over. Passwords often cannot match previous entries and involve multiple erroneous key elements to meet required fields. Working in a fast-paced ED, having to frequently create and update complicated passwords quickly transforms these safeguards into a barrier to use.

Finally, not all PDMPs track all schedule drugs. Schedule II drugs such as opioids have largely been the focus of PDMPs, but other drugs categorized in alternative schedules also have the potential for abuse. In 2011, for example, ED visits for benzodiazepine abuse, a schedule IV drug, was nearly equal to visits for opioids.^14^ ED records have demonstrated a strong association with benzodiazepine abuse and opioid abuse.^15^ Despite this potential for additional abuse, only 34 PDMPs monitor schedule II–V drugs.^16^ To address many of the usability limitations with PDMPs in EDs, we suggest several ways to optimize their implementation.

### Integration into ED Electronic Health Records

First, working to integrate the PDMPs with electronic health records (EHR) is a key to effectively using PDMPs. Currently, while clinicians are working in their hospital’s EHR system, most have to open a web-browser and log in to a separate, secure page with a separate username and password that is often a time-consuming process, further deterring the widespread use of the PDMP data. In the interest of time, physicians instead often resort to using prior EHR data to make determinations about possible drug seeking. Considering that EHR data is typically not shared between facilities, physicians base decisions on significantly smaller sample sizes. Indiana became the first state to merge an EHR with the PDMP. The integration was found to be highly effective with 58% of physicians prescribing fewer opioids or smaller quantities after the implementation of the PDMP data.^17^ Furthermore, integration of these systems could allow for improvements through unsolicited reporting “alerts” on the EHR for accessing physicians.^18^ These alerts could be used much like a “sepsis alert,” indicating a questionable prescription history of a patient immediately, allowing clinicians to further investigate if needed.^18^

### Unsolicited Reporting

Unsolicited reporting is a powerful tool through which PDMPs can automatically send alerts for drug-seeking behavior meeting a specific threshold to the appropriate authority. Such quantitative thresholds have already been implemented in several states with some success. In Virginia, thresholds for individuals receiving 10 prescriptions from 10 providers (10×10) or (15×15) within a six-month period were used.^19^ Subsequent periodic analysis of the data for automatic, unsolicited reporting showed a steady decrease in the number of individuals meeting thresholds, correlating to a decrease in likely diversion and abuse.^19^ Such automated reporting does come with risk as such policies raise concerns about patients being labeled an addict or postponing necessary treatment.^18^ In addition, physicians treating cancer patients or those requiring long-term opioid management have expressed concern for their reputation and licensure.^18^ However, in the context of the newly-approved National Quality Forum measures for limiting opioid prescribing, PDMPs can take such measures into consideration and would likely have an inverse effect by ensuring that guidelines are followed and patients are treated safely.^20^

### Providing Context for Opioid Prescriptions Through Data Analytics

Data analytics and data visualization may be ways to help contextualize opioid prescriptions for the busy EP. For example, by linking prescriptions to a particular diagnosis physicians may greatly reduce the guesswork involved for prescription behavior. At a glance, a patient with multiple prescriptions for both short- and long-acting, opioid pain medications may appear to be an opioid abuser. However, by tethering an explanatory diagnosis to such prescriptions, after investigation this patient could be found to have an extensive chronic pain condition warranting multiple prescriptions. Therefore, fewer mental resources may be required to rule out opioid abuse, reducing the potential for misinterpreting data and in turn provide quicker and better-informed emergency care.

### Expanding the Accessibility to PDMPs Within the ED

Another common complaint from attending physicians is the restriction allowing use of PDMPs only by licensed and practicing attending physicians, and excluding resident physicians. By allowing resident physicians access to PDMP information, the clinical care team could be more efficient particularly in academic settings where residents make many clinical decisions. In addition, allowing other providers who work in the ED, such as nurses and technicians, access to PDMP data may further amplify their effectiveness and use as a screening instead of a confirmation tool.

### Expanding the Scope of PDMPs

PDMPs have an extensive capability for tracking drug-seeking behavior and contacting the appropriate authorities such as prescribers, pharmacies, licensing boards or law enforcement. However, given that abuse is not limited to opioid misuse, but includes benzodiazepines and other schedule drugs as well, it is logical to assume that by extending PDMP records to include tracking of all scheduled drugs, PDMPs can have a greater impact against multiple forms of doctor shopping, drug abuse and diversion.

### Models of Well-designed PDMPs

Despite the issues highlighted above, some PDMPs studies still suggest a positive trend between their use and subsequent decreases in opioid and prescription-drug abuse. While opioid prescription overdose and abuse steadily increased from 2002–2010, by 2011 opioid prescriptions declined and consequently opioid overdose-related deaths fell and abuse plateaued.^21^ As more modern PDMP systems came online in conjunction with this decline, they are thought to have played a role in by reducing prescriptions in circulation and providing local governments with better resources to identify illicit activity. Furthermore, in Kentucky, Tennessee, New York and Ohio, early adopters of mandated PDMP use have shown preliminary results significant for a reduction in opioid prescribing as well as declines in multiple providers prescribing or in doctor shopping.^7^ Meanwhile, a 2016 study found a reduction on average of 1.12 fewer, opioid-related overdose deaths per 100,000 cases annually by implementing a PDMP program.^7^ Finally, another 2016 study showed that from 2003–2009, states without PDMPs experienced a steady increase in opioid exposures of 1.9% per quarter annually, while states with PDMPs in place experienced increases of only 0.2%.^2^

## CONCLUSION

EDs and their providers are on the front lines of the opioid crisis, treating significant portions of the surrounding community. As a result, their clinical decision-making has effects throughout their community, and improving the effectiveness of the PDMP in the ED has the potential to curb drug abuse and diversion. Yet PDMPs are currently complicated by myriad different strategies with varying state-to-state requirements and a lack of interconnectivity, which limit their usability and use. Several potential solutions exist to enhance PDMPs in the ED including integrating PDMPs with EHRs, implementing unsolicited reporting and prescription context, improving PDMP accessibility and data analytics, and expanding the scope of PDMPs.
